# Mediating effect of lower extremity muscle on the relationship between obesity and osteoarthritis in middle-aged and elderly women in Korea: based on the 2009-2011 Korea National Health and Nutrition Examination Survey

**DOI:** 10.4178/epih.e2024027

**Published:** 2024-02-02

**Authors:** Minjun Kim, Joonwoong Kim, Inhwan Lee

**Affiliations:** 1 Department of Physical Education, Yongin University, Yongin, Korea; 2 Department of Convergence, Seowon University, Cheongju, Korea; 3 Department of Smart Healthcare, Changwon National University, Changwon, Korea

**Keywords:** Body mass index, Knee osteoarthritis, Waist circumference, Lower extremity, Muscles, Abdominal obesity

## Abstract

**OBJECTIVES:**

This study investigated whether the lower extremity muscle mass index (LMI) mediates the relationship between general obesity, central obesity, and knee osteoarthritis in middle-aged and elderly women in Korea.

**METHODS:**

Data of 2,843 women aged ≥50 years were collected from the Korean National Health and Nutrition Examination Survey conducted between 2009 and 2011. General obesity and central obesity were evaluated based on body mass index (BMI) and waist circumference (WC), calculated through anthropometric measurements and body composition assessments. LMI was calculated by dividing the muscle mass in both legs—measured using the dual-energy X-ray absorptiometry—by body weight. Knee osteoarthritis was defined as a Kellgren-Lawrence scale (KL) grade of ≥2 as assessed through radiographic images.

**RESULTS:**

Knee osteoarthritis prevalence, indicated by KL grades, was significantly higher in the general obesity and central obesity groups compared to the normal group, and conversely, lower with varying LMI levels. Using mediation analysis with bootstrapping and adjusting for covariates, we found that LMI mediated the relationship between BMI and KL (β, 0.005; 95% confidence interval [CI], 0.000 to 0.010) and WC and KL grade (β, 0.002; 95% CI, 0.001 to 0.003), explaining 4.8% and 6.7% of the total effects of BMI and WC on KL grade, respectively.

**CONCLUSIONS:**

The study suggested that LMI partially mediates the link between general obesity and/or central obesity and knee osteoarthritis, proposing that a higher proportion of lower limb muscle mass relative to body weight can alleviate the increased risk of knee osteoarthritis caused by obesity.

## GRAPHICAL ABSTRACT

[Fig f3-epih-46-e2024027]

## Key Message

Research examining the mediating effect of lower extremity muscle mass on the relationships of knee osteoarthritis to general and central obesity is scarce. Herein, we demonstrate the mediating effect of lean mass index on the relationships of knee osteoarthritis with body mass index and waist circumference in Korean women aged 50 years or older. We propose that strengthening leg muscles may mitigate, to some extent, the risk of knee osteoarthritis associated with general and central obesity. We recommend a regimen for strengthening leg muscles through healthy lifestyle habits and regular strength training, in conjunction with weight and abdominal fat management.

## INTRODUCTION

Osteoarthritis (OA) is a prevalent joint disorder among the elderly, characterized by inflammation and pain resulting from progressive damage or degenerative changes in the cartilages that protect the joint. The most common site of OA is the knees [1,2]. Globally, knee osteoarthritis (KOA) is estimated to account for approximately 80% of OA disease burden [3]. Given the rapid aging of the population, the prevalence of KOA and the disease burden it imposes are expected to further escalate [4]. At present, no effective treatment exists for KOA, except for arthroplasty in an advanced stage [5]. However, this treatment carries a risk of postprocedure complications and incurs considerable health costs [6,7], making it critical to prevent the joint disorder before it progresses [4,7-9]. It is believed that early detection of modifiable risk factors that may affect the occurrence of KOA, such as lifestyle habits (e.g., sedentary lifestyle, malnutrition, and drinking) and health-related factors (e.g., high body-fat percentage, low muscle mass, and diseases), is an effective strategy for maintaining joints health from middle age onwards.

General obesity (GOB), defined as a body mass index (BMI; calculated using height and weight) of 25 kg/m^2^ or higher [10], is a well-known risk factor of KOA [11-15]. Currently, the worldwide prevalence of KOA is increasing linearly in proportion to the size of the obese population [16-19]. The pathogenesis of KOA due to obesity is the weight burden in the knees from excessive body weight, which functions as mechanical stress beyond the joints’ biological capacity, interferes with homeostasis, and causes structural damage, thereby accelerating disease progression [20]. Additionally, uncontrolled mechanical stress may facilitate the onset of KOA by causing synovitis through abnormal cellular activity [21,22].

Waist circumference (WC) is utilized as a primary index for abdominal obesity. In addition to GOB, central obesity (COB) defined based on WC, is reported to be a major risk factor for KOA [23-25]. A cohort study suggested that a high WC plays a causative role in the occurrence of KOA, with WC in a dose-response relationship with the risk for KOA [23]. In addition, COB is reported to increase the risk of early onset of KOA before age of 50 years and accelerate the manifestation of pain symptoms due to KOA [24].

Conversely, a sedentary lifestyle has been identified as a critical factor in reducing muscular strength and muscle mass [26]. The reduction in muscle mass causes GOB and COB by inducing physiological changes such as basal metabolism rate decrease and energy imbalance [27,28]. Given that weak muscular strength and low muscle mass in the lower extremities are reported to be another risk factor for the onset of KOA [29-32], lower extremity muscle mass (LMM) may mediate the effects of GOB and COB on KOA. Most previous studies, however, have been limited to simply testing the links of KOA to GOB, COB, and LMM, and research has rarely been conducted to examine the mediating effect of LMM on the relationships of KOA to GOB and COB. Accordingly, this study investigated whether or not LMM mediates the effects of GOB and COB on KOA in women of age 50 years or older (i.e., the age range in which KOA prevalence is high), in consideration of age and gender differences in KOA prevalence in Korea.

## MATERIALS AND METHODS

### Data source

In this study, the 2009-2011 Korea National Health and Nutrition Examination Survey (KNHANES) data were utilized. KNHANES is a nationwide survey annually conducted by the Korea Disease Control and Prevention Agency (KDCA, formerly Korea Centers for Disease Control and Prevention) to assess the citizens’ health and nutritional status with the purpose of creating healthrelated and nutrition-related policies. The sample was designed using a two-stage stratified cluster sampling method. The firststage stratification was based on the 2005 Population and Housing Census and the sampling was performed at the level of city/ province. The second-stage stratification sampling was based on gender and age. The survey data are available to the public on the KNHANES homepage, and details regarding the data, the survey design, etc. can be found on the web page presenting raw data (https://knhanes.kdca.go.kr/knhanes/sub03/sub03_01.do).

### Study participants

**Figure 1.**
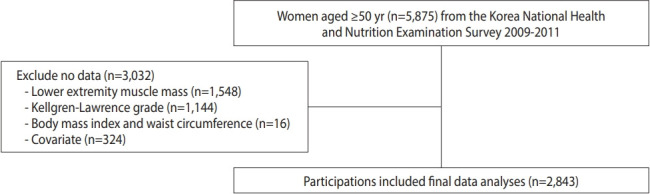
Flow chart of eligible participants in the study.

Initially, the study included 5,875 women aged ≥ 50 years who participated in the 4th phase or 5th phase of KNAHES (2009-2011), where in both body composition and osteoarthritis tests were performed. Of those, a total of 3,032 were excluded from the study owing to the following reasons: LMM was not assessed, n= 1,548; missing radiographic KOA image data, n= 1,144; missing BMI and WC data, n= 16; and missing data on the covariates, n= 324. Finally, data from 2,843 women were submitted for final analysis (Figure 1).

### Assessment of radiographic knee osteoarthritis

Radiographic images of bilateral weight-bearing knees from anterior-posterior and lateral views were obtained using SD 3000 Synchro Stand instrument (Accele Ray, Bern, Switzerland), with participants bending the knees by 30° (flexion). The images were examined by two radiologists using the Kellgren-Lawrence (KL) scale and for each participant, the agreed-upon grade was determined as the final severity level. If the disagreement exceeded one level, the higher grade was selected as the final grade. If two radiologists disagreed by more than one grade, a third radiologist was brought in to assess the image. The grade agreed upon by two of three radiologists was then determined as the final grade. Radiographic KOA was defined as a KL grade ≥ 2 [33].

### Assessment of anthropometric data and lower extremity muscle mass, general obesity, and abdominal obesity

The participants wore examination gowns while all anthropometric measurements were obtained. Height was measured using a Seca 225 stadiomete (Seca, Hamburg, Germany), and WC was measured at the level of the lowest rib and the center of the iliac crest by using an anthropometric tape measure. Body composition was assessed with a scan utilizing dual-energy X-ray absorptiometry (DXA) (Discovery-W fan-beam densitometer, Hologic, Bedford, MA, USA).

LMM was calculated by subtracting bone and fat masses from the lower extremity mass that was estimated via DXA. The calculated LMM was standardized to the lower extremity muscle mass index (LMI) using the equation, LMM/weight*100 [34]. Then, the LMI values were classified as low (lower 25%), moderate (middle 50%), and high (upper 25%).

BMI was calculated as weight (kg)/height (m^2^). Participants with a BMI of ≥ 25 kg/m^2^ or higher were classified as obese, in accordance with the criteria for obesity of the World Health Organization Regional Office for the Western-Pacific Region [10]. Additionally, COB was defined as a WC ≥ 85 cm, in accordance with the Korean Society for the Study of Obesity’s criterion for COB [35].

### Covariates

Covariates were age, average monthly household income, education, marital status, employment status, region, smoking, binge alcohol, inactivity, energy intake, protein intake, self-reported health status, menopause, and comorbidity. Data on the covariates were obtained using self-administered questionnaires. Average monthly household income was measured in the unit of 10,000 Korean won. Education level was categorized as “lower than elementary school,” “middle/high school,” and “college or higher.” Marital status was categorized as “married” and “widowed/divorced/unmarried.” Employment status was classified according to current job status to earn money, and the region was categorized as “urban” and “rural.” Smoking was defined as having a lifetime smoking history of 100 or more cigarettes or currently smoking [36], and binge alcohol as having 5 or more glasses of alcohol per session [37]. Inactivity was defined as a lack of moderate-to-intense physical activity per week [38]. Energy intake and protein intake were assessed by examining daily consumptions in g/day. Self-reported health status was classified to “good or very good,” “fair,” and “poor or very poor.” Regarding comorbidity, the number of physician-diagnosed conditions out of hypertension, hypercholesterolemia, diabetes, stroke, angina pectoris, and myocardial infarction was categorized as 0, 1, and ≥ 2.

### Statistical analysis

In all data, continuous variables are presented with means and standard deviations, and categorical variables are presented with frequencies and percentages (%) of each category. To compare continuous and categorical variables according to the presence or absence of KOA, GOB, and COB, independent samples t-test and chi-square test were performed, respectively. Polynomial contrast in one-way analysis of variance and linear-by-linear association in cross-tab analysis were used to test for trends according to the LMI categories. Additionally, mediation analysis using bootstrapping (n= 10,000, a method of utilizing random re-sampling and iterating the test) was performed to investigate the mediating effect of LMI on the relationships between BMI and KL grade and between WC and KL grade. All statistical analyses were conducted using SPSS version 29.0 (IBM Corp., Armonk, NY, USA). To additionally perform mediation analysis, the PROCESS macro developed by Andrew F. Hayes was installed in SPSS. Statistical significance in hypothesis testing was set at α= 0.05.

### Ethics statement

The Korea National Health and Nutrition Examination Survey was approved by the Korea Centers for Disease Control and Prevention’s Institutional Review Board (reference No. 2009-01CON03-2C, 2010-02CON-21-C, 2011-02CON-06-C), and participants provided written informed consent to participate in this study.

## RESULTS

### Comparisons between the presence and absence of knee osteoarthritis

Table 1 shows the results of analyses comparing variables between groups with and without KOA (KOA and non-KOA groups, respectively). In the KOA group, age (p< 0.001), weight (p< 0.001), BMI (p< 0.001), WC (p< 0.001), body fat (p< 0.001), the proportion of participants with lower education level (p< 0.001), proportion of participants who self-reported their health status as other than “good or very good” (p< 0.001), proportion of participants experiencing menopause (p< 0.001), and proportion of participants with ≥ 2 chronic diseases (p< 0.001) were significantly higher than that in the non-KOA group. Additionally, height (p< 0.001), LMI (p< 0.001), average monthly household income (p< 0.001), energy intake (p< 0.001), protein intake (p< 0.001), and the proportions of married participants (p< 0.001) and participants living in urban areas (p< 0.001) were significantly lower in KOA group than that in the non-KOA group.

### Comparisons between the presence and absence of general obesity and central obesity

Table 2 shows the results of analyses comparing variables between the groups with and without GOB and between the groups with and without COB. First, in the GOB group, WC (p< 0.001), weight (p< 0.001), LMM (p< 0.001), body fat (p< 0.001), KL grade (p< 0.001), and the proportion of participants with lower education (p<0.001), proportion of participants binge alcohol (p=0.029), proportion of participants with ≥ 2 chronic diseases (p< 0.001), and proportion of participants with KOA (p< 0.001) were significantly higher than that in the non-GOB group. Furthermore, LMI (p< 0.001), average monthly household income (p= 0.016), and the proportions of smokers (p= 0.014) and participants with inactivity (p= 0.003) were significantly lower in the GOB group than that in the non-GOB group.

The results of analyses comparing the groups with and without COB were as follows. In the COB group, BMI (p < 0.001), age (p< 0.001), weight (p< 0.001), height (p< 0.001), LMM (p< 0.001), body fat (p< 0.001), KL grade (p< 0.001), and the proportion of participants with lower education (p< 0.001), proportion of participants with binge alcohol (p= 0.010), proportion of participants whose self-reported health status was “poor or very poor” (p<0.001), proportion of participants with ≥ 2 chronic diseases (p< 0.001), and proportion of participants with KOA (p< 0.001) were significantly higher than that in the non-COB group. Additionally, LMI (p< 0.001), average monthly household income (p= 0.016), protein intake (p= 0.011), and the proportion of married participants (p= 0.001) were significantly lower in the COB group than that in the non-COB group.

### Trend analysis according to lower extremity muscle mass index

Table 3 shows the results of the trend analysis according to LMI. As LMI increased, the following variables showed decreasing trends: age (p< 0.001), height (p< 0.001), BMI (p< 0.001), WC (p < 0.001), body fat (p < 0.001), KL grade (p < 0.001), and the proportion of participants with lower education (p< 0.001), the proportion of married participants (p= 0.007), the proportion of participants living in urban area (p = 0.017), the proportion of participants whose self-reported health status was other than “good or very good” (p= 0.008), those with 2 or more chronic diseases (p< 0.001), and those with KOA (p< 0.001). In contrast, as LMI increased, height (p< 0.001), LMM (p< 0.001), and energy intake (p< 0.001) showed increasing trends.

### Analysis of the mediating effect of lower extremity muscle mass index on the relationships of Kellgren-Lawrence grade with body mass index and waist circumference

**Figure 2.**
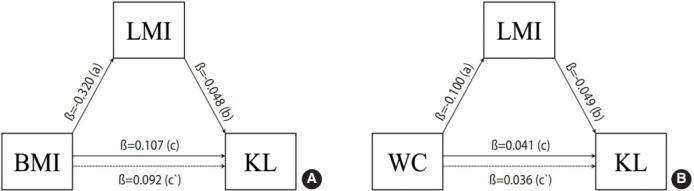
Mediation analysis. Path coefficients of body mass index (BMI; A) or waist circumference (WC; B) on Kellgren-Lawrence grade (KL) through lower extremity muscle mass index (LMI). Path a: estimated coefficient for the regression with BMI (A) or WC (B) predicting LMI; Path b: estimated coefficient for the regression with LMI predicting KL; Path c=total effects for the regression with BMI (A) or WC (B) predicting KL; Path c`=direct effects for the regression with BMI (A) or WC (B) predicting KL independent of LMI.

Figure 2A and Table 4 present the relationships between BMI and KL grade and between WC and KL grade, both mediated by LMI. First, the analysis to evaluate the mediating effect of LMI on the relationship between BMI and KL grade revealed that BMI had a direct effect on KL grade (ß, 0.092; p< 0.001: c` path), and additionally, it had an indirect effect on KL grade via LMI (Figure 2A and Table 4). BMI had a negative relationship with LMI (ß, -0.320; p< 0.001: a path), which was negatively associated with KL grade (ß, -0.048; p= 0.001: b path). Even after the model was adjusted for all covariates, BMI and LMI were significant predictors of KL grade (ß, -0.032; p= 0.046: b path; ß, 0.100; p< 0.001: c` path).

In bootstrap analysis of the mediating effect of LMI on the relationship between BMI and KL grade, the 95% confidence interval (CI, 0.006 to 0.025) did not include 0, suggesting that the relationship between BMI and KL grade was mediated by LMI. LMI explained 14.0% of the total effect of BMI on KL grade. The mediating effect of LMI was significant even after the model was adjusted for covariates (95% CI, 0.000 to 0.010), and of the total effect in the adjusted model, 4.8% was explained via LMI. These results suggest that while GOB had a direct effect on the occurrence of KOA, it also indirectly influenced KOA through leg muscles.

Likewise, the analysis of the mediating effect of LMI on the relationship between WC and KL grade showed that WC had a direct effect on KL grade (ß, 0.036; p< 0.001: c` path), and also, indirectly influenced the grade via LMI (Figure 2B and Table 4). WC had a negative relationship with LMI (ß, -0.100; p< 0.001: a path), which was negatively related to KL grade (ß, -0.049; p= 0.001: b path). Even after adjusting for covariates, WC and LMI were significant predictors of KL grade (ß, -0.049; p= 0.002: b path; ß, 0.028; p< 0.001: c` path).

In bootstrap analysis of the mediating effect of LMI on the relationship between WC and KL grade, the 95% CI (0.002 to 0.008) did not include 0, suggesting that LMI intervened in the relationship between WC and KL grade. LMI explained 12.2% of the total effect of WC on KL grade. Even after adjusting for covariates, the mediating effect of LMI on the relationship between WC and KL grade was significant (95% CI, 0.001 to 0.003) and 6.7% of the total effect was explained via LMI. The results suggest that while COB had a direct effect on the onset of KOA, it also indirectly influenced KOA via leg muscles.

## DISCUSSION

This study investigated the mediating effect of LMI on the relationships of KOA with GOB and COB in middle-aged and elderly women (aged ≥ 50 years) in Korea. Our findings revealed that BMI (based on which GOB was defined), WC (based on which COB was defined), and LMI all were significant predictors of KL grade (based on which KOA was diagnosed). In addition, the effects of BMI and WC on KL grade were partially mediated by LMI.

Obesity overloads the knees, producing excessive stress in the joints; inducing pathological damages in the cartilages [39-42], osteochondral interface [43], and meniscus; and expediting the occurrence of KOA [44]. Additionally, it is reported that some proinflammatory cytokines produced in adipose tissue induce the metabolic process of joint breakdown, promoting KOA [45]. GOB and COB, characterized by overweight and abnormal accumulation of fat, are known to play a causative role in the occurrence of KOA. That they are primary risk factors for KOA, regardless of race, gender, and age [11-15,23-25], is an established fact in epidemiology. The current finding that KL grade was positively associated with BMI and WC at a significant level is consistent with the findings of previous studies.

Leg muscles generate power to respond to external loads, absorbing the burden on the knee loads and stabilizing the joints in dynamic situations [20]. In experimental studies that assessed the contribution of leg muscles to knee stability, a protective effect was found in specific muscles surrounding the joints [46], and furthermore, most muscles in the legs contributed to knee stability in a variety of ways [47]. These findings suggest that in general, the stronger the leg muscles, the stronger the protective effect against KOA. Hence, given that muscular strength is proportional to muscle mass, it is reasoned that LMM reflects the level of strength of leg muscles [48] and that a protective effect against KOA is provided by LMM as well as leg muscle strength. Indeed, this line of thought is supported by the finding that LMI (LMM/weight) was negatively associated with the risk of KOA at a significant level in representative samples of Korean adult population [31,32]. A cohort study conducted by Segal et al. [49], however, did not find a protective effect against KOA in thigh muscle mass. The conflicting results in the current and the previous studies are speculated to be because absolute LMM value was used in one study, whereas its relative value, with body weight taken into account, was used in the other. Considering that generally, body composition measures like body fat and muscle mass are proportional to body weight, it is anticipated that the relative LMM value adjusted for the effect of weight will more accurately reflect leg muscle strength. In the current study, LMI and KL grade showed a significant negative relationship, which was in line with the previous finding that LMI—in which LMM was adjusted for body weight—had a protective effect against KOA [31,32]. In summary, these findings suggest that greater LMM relative to body weight may have a protective effect against KOA.

This is the first study to report that the relationships between KL grade and BMI and between KL grade and WC were partially mediated by LMI. The partial mediating effect of LMI was significant irrespective of other potential covariates like demographic, socioeconomic, and health-related variables. Similarly, Kim et al. [31] analyzed data from 4,194 participants of the 2010-2011 KNHANES and found that as LMM increased, the risk for KOA decreased linearly in both participants with normal-weight and those with obesity. In the current study too, the risk for KOA was significantly lower in the large vs. small LMM group, regardless of all other covariates [31]. The previous and present study findings suggest that strengthening the leg muscles may reduce the impact of GOB and COB on KOA and that KOA prevention strategy should include the strengthening of leg muscles with healthy lifestyle habits and regular weight training. However, the mediating effect of LMI on the relationships of KOA with BMI and WC was small (4.6 and 6.5%, respectively). Based on this finding, it is believed that for the prevention of KOA, weight and abdominal fat management should take priority over strengthening of leg muscles.

On the basis of the current finding regarding the mediating effect of LMI on the relationships of KOA with GOB and COB, we hypothesize that LMI may provide a protective effect against the occurrence of KOA owing to GOB and COB, as explained in the following discussion. From a biomechanical perspective, GOB and COB destabilize the knees by exerting abnormal levels of stress on the joints. In contrast, muscles absorb shock delivered to joints, contributing to joint stability [20]. Therefore, it is expected that leg muscles buffer the impact of abnormal stress levels at the knee joints due to GOB or COB. This discussion is supported by the current study finding as well as a previous finding that the risk for KOA was higher in sarcopenic obesity (a condition in which obesity is accompanied by sarcopenia) than that in obesity alone [50].

The current study has a few limitations. First, the findings may vary among different races, age groups, and genders. In this study, participants were limited to women of age ≥ 50 years, while women of age ≤ 49 years and men of any age were excluded on the basis of low KOA prevalences. Accordingly, the current study findings may not be generalizable to Korean men and women of age ≤ 49 years, or other ethnic groups. Second, information regarding confounding factors that may affect KOA (e.g., medications, metabolic risk factors, hormones, etc.) is not provided in KNHANES, and therefore, the possibility that results may be influenced by potential, unexamined covariates cannot be excluded. Third, information regarding the history of arthroplasty is not provided in KNHANES. Hence, the possibility that those who underwent the procedure were included in the study sample cannot be excluded. Fourth, this study was a cross-sectional study and cannot explain causation. Finally, this study does not provide a physiological mechanism of LMI’s mediating effect on the relationships between GOB and KOA and between COB and KOA, and additional research is necessary.

To conclude, this study demonstrated the mediating effect of LMI on the relationships of KOA with BMI and WC in Korean women aged 50 years or older. Our findings suggest that strengthening leg muscles may reduce, to some extent, the risk of KOA owing to GOB and COB. As a prevention strategy for KOA, strengthening the muscles through healthy lifestyle habits and regular strength training in combination with weight and abdominal fat managements should be recommended.

## Figures and Tables

**Figure f3-epih-46-e2024027:**
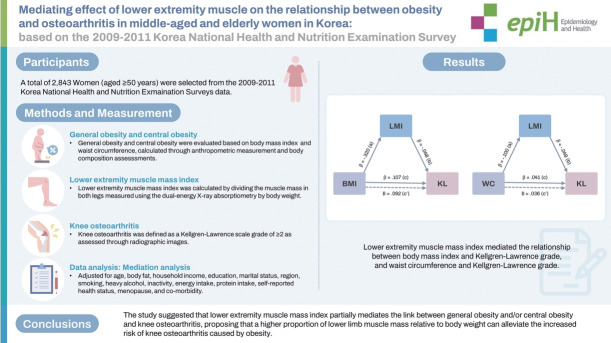


**Table 1.** Descriptive statistics of measured parameters according to KOA

Variables	Total (n=2,843)	Normal (n=1,477)	KOA (n=1,366)	p-value
KL grade	1.67±1.44	0.42±0.49	3.02±0.75	<0.001
Age (yr)	63.8±9.0	60.4±8.3	67.4±8.3	<0.001
Body weight (kg)	57.0±8.7	56.0±8.1	58.1±9.3	<0.001
Height (cm)	153.2±5.8	154.2±5.6	152.2±5.9	<0.001
LMM (kg)	11.3±1.6	11.2±1.6	11.3±1.7	0.621
LMI (%)	20.0±2.0	20.3±2.0	19.7±2.0	<0.001
BMI (kg/m^2^)	24.3±3.3	23.6±3.0	25.0±3.3	<0.001
WC (cm)	82.3±9.3	80.2±8.7	84.7±9.3	<0.001
Body fat (%)	34.7±5.5	34.0±5.4	35.4±5.5	<0.001
Socioeconomic status				
Household income (10,000 KRW/mo)	140.7±246.4	164.5±270.4	115.0±214.6	<0.001
Education				<0.001
Lower than elementary school	1,822 (64.1)	785 (53.1)	1,037 (75.9)	
Middle/high school	886 (31.2)	588 (39.8)	298 (21.8)	
Over than college	135 (4.7)	104 (7.1)	31 (2.3)	
Marital status				<0.001
Married	1,923 (67.6)	1,123 (76.0)	800 (58.6)	
Widowed/divorced/unmarried	920 (32.4)	354 (24.0)	566 (41.4)	
Region of residence				<0.001
Urban	1,947 (68.5)	1,089 (73.7)	858 (62.8)	
Rural	896 (31.5)	388 (26.3)	508 (37.2)	
Health-related parameters				
Smoking	195 (6.9)	100 (6.8)	95 (7.0)	0.846
Binge alcohol	106 (3.7)	54 (3.7)	52 (3.8)	0.832
Inactivity	2,265 (79.7)	1,166 (78.9)	1,099 (80.5)	0.318
Energy intake (g/day)	1,591.1±615.2	1,640.5±615.4	1,537.7±610.7	<0.001
Protein intake (g/day)	53.0±27.6	56.1±29.3	49.6±25.1	<0.001
Self-reported health status				<0.001
Good or very good	765 (26.9)	410 (27.8)	355 (26.0)	
Fair	1,074 (37.8)	607 (41.1)	467 (34.2)	
Poor or very poor	1,004 (35.3)	460 (31.1)	544 (39.8)	
Menopause	2,684 (94.4)	1,353 (91.6)	1,331 (97.4)	<0.001
Comorbidity				<0.001
0	1,305 (45.9)	797 (54.0)	508 (37.2)	
1	932 (32.8)	419 (28.4)	513 (37.6)	
≥2	606 (21.3)	261 (17.6)	345 (25.3)	

Values are presented as mean±standard deviation or number (%). KOA, knee osteoarthritis; KL, Kellgren-Lawrence; LMM, lower extremity muscle mass; LMI, lower extremity muscle mass index; BMI, body mass index; WC, waist circumference; KRW, Korean won.

**Table 2.** Descriptive statistics of measured parameters according to GOB and COB

Variables	BMI (kg/m^2^)		p-value	WC (cm)		p-value
	<25 (n=1,763)	≥25 (n=1,080)		<85 (n=1,759)	≥85 (n=1,084)	
BMI (kg/m^2^)	22.3±1.9	27.5±2.2	<0.001	22.6±2.3	27.0±2.7	<0.001
WC (cm)	77.5±6.8	90.2±7.0	<0.001	76.6±5.8	91.6±5.6	<0.001
Age (yr)	63.8±9.4	63.7±8.4	0.646	63.1±9.3	64.8±8.4	<0.001
Body weight (kg)	52.5±6.1	64.5±7.0	<0.001	52.9±6.5	63.8±7.6	<0.001
Height (cm)	153.4±6.0	153.0±5.6	0.115	152.9±5.9	153.8±5.7	<0.001
LMM (kg)	10.7±1.4	12.1±1.6	<0.001	10.7±1.4	12.1±1.6	<0.001
LMI (%)	20.6±2.0	19.0±1.7	<0.001	20.5±2.0	19.1±1.8	<0.001
Body fat (%)	32.6±5.2	38.1±4.0	<0.001	32.8±5.3	37.7±4.2	<0.001
Socioeconomic status						
Household income (10,000 KRW/mo)	148.3±256.5	128.4±228.5	0.016	149.2±255.8	126.9±229.8	0.016
Education			<0.001			<0.001
Lower than elementary school	1,082 (61.3)	740 (68.5)		1,040 (59.1)	782 (72.1)	
Middle/high school	581 (33.0)	305 (28.3)		611 (34.8)	275 (25.4)	
Over than college	100 (5.7)	35 (3.2)		108 (6.1)	27 (2.5)	
Marital status			0.482			<0.001
Married	1,201 (68.1)	772 (66.9)		1,233 (70.1)	690 (63.7)	
Widowed/divorced/unmarried	562 (31.9)	358 (33.1)		526 (29.9)	394 (36.3)	
Region of residence			0.975			0.090
Urban	1,207 (68.5)	740 (68.5)		1,225 (69.6)	722 (66.6)	
Rural	556 (31.5)	340 (31.5)		534 (30.4)	362 (33.4)	
Health-related parameters						
Smoking	137 (7.8)	58 (5.4)	0.014	125 (7.1)	70 (6.5)	0.506
Binge alcohol	55 (3.1)	51 (4.7)	0.029	53 (3.0)	53 (4.9)	0.010
Inactivity	1,436 (81.5)	829 (76.8)	0.003	1,406 (79.9)	859 (79.2)	0.658
Energy intake (g/day)	1,586.1±638.9	1,599.2±574.5	0.582	1,598.8±637.9	1,578.7±576.4	0.398
Protein intake (g/day)	53.1±27.9	52.8±27.0	0.830	54.0±28.9	51.4±25.2	0.011
Self-reported health status			0.125			<0.001
Good or very good	480 (27.2)	285 (26.4)		501 (28.5)	264 (24.4)	
Fair	685 (38.9)	389 (36.0)		697 (39.6)	377 (34.8)	
Poor or very poor	598 (33.9)	406 (37.6)		561 (31.9)	443 (40.8)	
Menopause	1,670 (94.7)	1,014 (93.9)	0.346	1,650 (93.8)	1,034 (95.4)	0.074
Comorbidity			<0.001			<0.001
0	921 (52.2)	384 (35.5)		948 (53.9)	357 (32.9)	
1	549 (31.1)	383 (35.5)		541 (30.8)	391 (36.1)	
≥2	293 (16.7)	313 (29.0)		270 (15.3)	336 (31.0)	
KL grade	1.43±1.40	2.06±1.44	<0.001	1.41±1.39	2.08±1.43	<0.001
Radiographic KOA	712 (40.4)	654 (60.6)	<0.001	700 (39.8)	666 (61.4)	<0.001

Values are presented as mean±standard deviation or number (%). GOB, general obesity; COB, central obesity; BMI, body mass index; WC, waist circumference; LMM, lower extremity muscle mass; LMI, lower extremity muscle mass index; KRW, Korean won; KL, Kellgren-Lawrence; KOA, knee osteoarthritis.

**Table 3.** Descriptive statistics of measured parameters according to LMI

Variables	Lower LMI (n=711, 25.0%)	Middle LMI (n=1,421, 50.0%)	Upper LMI (n=711, 25.0%)	p for trend
LMI (%)	17.5±0.9	19.9±0.7	22.7±1.2	<0.001
Age (yr)	65.5±8.8	63.2±8.8	63.4±9.4	<0.001
Body weight (kg)	60.7±9.1	57.4±8.0	52.7±7.8	<0.001
Height (cm)	151.8±5.5	153.5±5.7	154.1±6.3	<0.001
LMM (kg)	10.6±1.6	11.3±1.6	11.8±1.6	<0.001
BMI (kg/m^2^)	26.3±3.4	24.3±2.8	22.1±2.6	<0.001
WC (cm)	87.5±9.4	82.4±8.2	76.9±8.0	<0.001
Body fat (%)	40.2±3.4	34.9±3.2	28.7±4.6	<0.001
Socioeconomic status				
Household income (10,000 KRW/mo)	240.1±9.0	230.1±6.1	281.3±10.5	0.238
Education				<0.001
Lower than elementary school	504 (70.9)	890 (62.6)	428 (60.2)	
Middle/high school	185 (26.0)	462 (32.5)	239 (33.6)	
Over than college	22 (3.1)	69 (4.9)	44 (6.2)	
Marital status				0.007
Married	438 (61.6)	999 (70.3)	486 (68.4)	
Widowed/divorced/unmarried	273 (38.4)	422 (29.7)	225 (31.6)	
Region of residence				0.017
Urban	489 (68.8)	1,011 (71.1)	447 (62.9)	
Rural	222 (31.2)	410 (28.9)	264 (37.1)	
Health-related parameters				
Smoking	42 (5.9)	98 (6.9)	55 (7.7)	0.173
Binge alcohol	28 (3.9)	54 (3.8)	24 (3.4)	0.576
Inactivity	572 (80.5)	1,141 (80.3)	552 (77.6)	0.188
Energy intake (g/day)	1,508.7±556.2	1,607.2±631.5	1,641.4±630.9	<0.001
Protein intake (g/day)	51.0±28.1	53.7±26.9	53.6±28.2	0.075
Self-reported health status				0.008
Good or very good	171 (24.1)	397 (27.9)	197 (27.7)	
Fair	246 (34.6)	556 (39.1)	272 (38.3)	
Poor or very poor	294 (41.3)	468 (33.0)	242 (34.0)	
Menopause	687 (96.6)	1,325 (93.2)	672 (94.5)	0.083
Comorbidity				<0.001
0	249 (35.0)	630 (44.3)	426 (59.9)	
1	249 (35.0)	487 (34.3)	196 (27.6)	
≥2	213 (30.0)	304 (21.4)	89 (12.5)	
KL grade	1.52±0.06	1.41±0.04	1.34±0.05	<0.001
Radiographic KOA	423 (59.5)	660 (46.4)	283 (39.8)	<0.001

Values are presented as mean±standard deviation or number (%). LMI, lower extremity muscle mass index; LMM, lower extremity muscle mass; BMI, body mass index; WC, waist circumference; KRW, Korean won; KL, Kellgren-Lawrence; KOA, knee osteoarthritis.

**Table 4.** The association between obesity and KL, mediated by LMI, in Korean women aged over 50 years^1^

Path^2^	Model 1				Model 2			
	ß (SE)	95% CI		p-value	ß (SE)	95% CI		p-value
		UL	LL			UL	LL	
General obesity								
BMI→LMI, a	-0.320 (0.010)	-0.340	-0.300	<0.001	-0.147 (0.010)	-0.166	-0.127	<0.001
LMI→KL, b	-0.048 (0.015)	-0.077	-0.019	0.001	-0.032 (0.016)	-0.064	-0.001	0.046
Total effect, c	0.107 (0.008)	0.092	0.123	<0.001	0.105 (0.009)	0.088	0.122	<0.001
Direct effect, c`	0.092 (0.009)	0.074	0.111	<0.001	0.100 (0.009)	0.082	0.118	<0.001
Indirect effect, ab	0.015 (0.005)	0.006	0.025		0.005 (0.003)	0.000	0.010	
Indirect to total effect (%)	14.0				4.8			
Central obesity								
WC→LMI, a	-0.100 (0.004)	-0.107	-0.093	<0.001	-0.040 (0.004)	-0.047	-0.033	<0.001
LMI→KL, b	-0.049 (0.014)	-0.077	-0.021	0.001	-0.049 (0.016)	-0.081	-0.017	0.002
Total effect, c	0.041 (0.003)	0.035	0.046	<0.001	0.030 (0.003)	0.024	0.036	<0.001
Direct effect, c`	0.036 (0.003)	0.030	0.042	<0.001	0.028 (0.003)	0.022	0.034	<0.001
Indirect effect, ab	0.005 (0.001)	0.002	0.008		0.002 (0.001)	0.001	0.003	
Indirect to total effect (%)	12.2				6.7			

KL, Kellgren-Lawrence grade; LMI, lower extremity muscle mass index; SE, standard error; CI, confidence interval; UL, upper limit; LL, lower limit; BMI, body mass index; WC, waist circumference.

1 Model 1: Non-adjusted; Model 2: Adjusted for age, body fat, household income, education, marital status, region, smoking, binge alcohol, inactivity, energy intake, protein intake, self-reported health status, menopause, comorbidity.

2 In the mediation model, the indirect effect is the product of path coefficients a (BMI or WC→LMI) and b (LMI→KL); The direct effect is the coefficient c`; The total effect (c) is equal to the sum of the direct and indirect (c`+ ab).

## Supplementary materials

Korean version is available at https://doi.org/10.4178/epih.e2024027.
