# Supplementary material for: Mediating effect of lower extremity muscle on the relationship between obesity and osteoarthritis in middle-aged and elderly women in Korea: based on the 2009-2011 Korea National Health and Nutrition Examination Survey
Source: Epidemiol Health. 2024 Feb 2;46:e2024027. doi: 10.4178/epih.e2024027 (PMC11099567; doi:10.4178/epih.e2024027)
Supplement: Supplementary file 1 [file epih-46-e2024027-Korean-Supplementary.pdf]

Open Access

Volume: 46, Article ID: e2024027
https://doi.org/10.4178/epih.e2024027

epiH Epidemiology and Health

ORIGINAL ARTICLE

한국 중·고령 여성들의 비만과 무릎 골관절염의 연관성에 대한 다리근육의 매개효과 분석

김민준[1], 김준웅[2], 이인환[3]

[1]용인대학교 체육학과, [2]서원대학교 융복합학과, [3]창원대학교 스마트헬스케어학과

Mediating effect of lower extremity muscle on the relationship between obesity and osteoarthritis in middle-aged and elderly women in Korea: Based on the 2009-2011 Korea National Health and Nutrition Examination Survey

Minjun Kim[1], Joonwoong Kim[2], Inhwan Lee[3]

[1]*Department of Physical Education, Yongin University, Yongin, Korea;* [2]*Department of Convergence, Seowon University, Cheongju, Korea;* [3]*Department of Smart Healthcare, Changwon National University, Changwon, Korea*

OBJECTIVES: This study investigated whether the lower extremity muscle mass index (LMI) mediates the relationship between general obesity, central obesity, and knee osteoarthritis in middle-aged and elderly women in Korea.
METHODS: Data of 2,843 women aged ≥50 years were collected from the Korean National Health and Nutrition Examination Survey conducted between 2009 and 2011. General obesity and central obesity were evaluated based on body mass index (BMI) and waist circumference (WC), calculated through anthropometric measurements and body composition assessments. LMI was calculated by dividing the muscle mass in both legs—measured using the dual-energy X-ray absorptiometry—by body weight. Knee osteoarthritis was defined as a Kellgren-Lawrence scale (KL) grade of ≥2 as assessed through radiographic images.
RESULTS: Knee osteoarthritis prevalence, indicated by KL grades, was significantly higher in the general obesity and central obesity groups compared to the normal group, and conversely, lower with varying LMI levels. Using mediation analysis with bootstrapping and adjusting for covariates, we found that LMI mediated the relationship between BMI and KL (β, 0.005; 95% confidence interval [CI], 0.000 to 0.010) and WC and KL grade (β, 0.002; 95% CI, 0.001 to 0.003), explaining 4.8% and 6.7% of the total effects of BMI and WC on KL grade, respectively.
CONCLUSIONS: The study suggested that LMI partially mediates the link between general obesity and/or central obesity and knee osteoarthritis, proposing that a higher proportion of lower limb muscle mass relative to body weight can alleviate the increased risk of knee osteoarthritis caused by obesity.

KEY WORDS: Body mass index, Knee osteoarthritis, Waist circumference, Lower extremity, Muscles, Abdominal obesity

Correspondence: Inhwan Lee
Department of Smart Healthcare, Changwon National University, Changwondaehak-ro, Uichang-gu, Changwon 51140, Korea
E-mail: ansh00@changwon.ac.kr
Received: Oct 20, 2023 / Accepted: Jan 8, 2024 / Published: Feb 2, 2024

This article is available from: https://e-epih.org/

This is an open-access article distributed under the terms of the Creative Commons Attribution License (https://creativecommons.org/licenses/by/4.0/), which permits unrestricted use, distribution, and reproduction in any medium, provided the original work is properly cited.

© 2024, Korean Society of Epidemiology

서 론

골관절염(osteoarthritis, OA)은 관절을 보호하고 있는 연골의 점진적인 손상이나 퇴행성 변화로 인해 관절 주변 조직에 손상이 생겨 염증과 통증이 발생하는 노인 인구에서 흔한 관절 질환이며, 무릎에서 가장 빈번하게 발생한다[1,2]. 전 세계적으로 무릎 골관절염(knee osteoarthritis, KOA)은 OA로 인한 질병 부담의 약 80%를 차지하는 것으로 추정되며[3], 인구 고령화 속도를 감안할

때, 향후 KOA의 유병률과 이로 인한 질병 부담은 더욱 가중될 것으로 예견된다[4]. 현 시점에서 KOA가 진행된 단계에서 관절성형술(arthroplasty)을 제외하고 마땅한 치료법이 없으며[5], 이마저도 수술 후 부작용의 위험이 있고 상당한 건강관리 비용(health costs)을 발생시킨다는 점을 고려할 때[6,7], 질환의 진행 전 이를 예방하는 것이 매우 중요하며[4,7-9], 신체활동 부족, 영양 불균형, 음주 등을 포함한 생활습관 요인, 높은 체지방, 낮은 근육량, 질병 등을 포함한 건강관련요인 등 KOA 발병에 영향을 미칠 수 있는 수정가능인자를 조기에 식별하는 것은 중년 이후 관절 건강을 유지할 수 있는 효과적 전략이 될 것으로 판단된다.

신장과 체중에 근거한 체질량지수(body mass index, BMI)가 25 kg/m^2 이상에 해당하는 것으로 정의되는 비만(general obesity, GOB) [10]은 KOA의 대표적인 위험 요인으로 알려져 있으며 [11-15], 현재, 전 세계적으로 KOA의 유병률은 GOB 인구와 비례하여 증가하는 선형 추세를 나타내고 있다[16-19]. 병인학적으로 GOB로 인한 KOA의 발병은 과도한 체중으로 무릎 관절에 가해지는 하중 부담에 기인하며, 이는 무릎 관절이 감당할 수 있는 생물학적 능력을 초과하는 기계적 스트레스로 작용하여 관절의 항상성을 무너뜨리고, 구조적 손상을 야기하여 KOA의 발병 진행을 가속화한다[20]. 또한 과도한 기계적 스트레스는 비정상적인 세포 활동을 통해 활막염을 유발하여 KOA의 발병을 촉진하기도 한다[21,22].

허리둘레(waist circumference, WC)는 복부비만(central obesity, COB)을 평가하는 대표적인 지표로 활용되며, WC에 근거한 COB는 GOB와 더불어 KOA의 주요 위험 요인으로 보고된다[23-25]. 이와 관련한 코호트 연구에 따르면 높은 WC는 KOA의 발병에 인과적 역할을 하며, WC와 KOA의 발병 위험은 용량 반응 관계(dose response)를 나타낸다[23]. 또한 COB는 50세 이전 KOA의 조기 발병 위험을 증가시키고, 이로 인한 통증의 조기 발현을 촉진하는 것으로도 보고된다[24].

한편, 신체활동 부족은 근력 및 근육량 감소의 주요 인자로 보고되며[26], 근육량 감소는 기초대사량 저하, 에너지 불균형 등의 생리적 변화를 초래하여 GOB 및 COB를 유발하는 것으로 보고된다[27,28]. 이러한 관점에서 낮은 다리의 근력과 근육이 KOA의 발병 위험을 증가시키는 또 다른 위험 요인으로 보고됨[29-32]을 감안할 때, 다리근육량(lower extremity muscle mass, LMM)이 KOA에 대한 GOB와 COB의 영향을 중재할 가능성이 있다. 그러나 현재까지 진행된 선행연구의 대부분은 KOA와 GOB 및 COB, LMM의 단편적인 연관성 검증에 국한되며, GOB 및 COB와 KOA의 관계에서 LMM의 매개 효과를 조사한 연구는 전무한 실정이다. 이에 본 연구는 한국의 KOA 유병률에 대한 연령 및 성별 차이를 고려하여 유병률이 높은 50세 이상의 중·고령 여성을 대상으로 LMM이 KOA에 대한 GOB 및 COB의 영향을 매개하는지 여부를 조사하였다.

연구방법

자료 수집

본 연구는 질병관리청에서 우리나라 국민의 건강 및 영양상태를 평가하고 이에 대한 정책수립을 목적으로 2005년부터 매년 실시하고 있는 국민건강영양조사의 2009-2011년 자료를 이용하였다. 본 연구의 조사 대상자 설계는 2단계 층화집락추출법을 이용하였으며, 2005년 인구주택 총 조사의 내용에 근거하여 1단계 층화는 시도별에 기반하였고, 2단계 층화는 성별, 연령에 근거하여 조사대상 선정이 이루어졌다. 또한, 본 조사의 데이터는 국민건강영양조사 홈페이지에서 개방 형태로 제공되고 있으며, 관련 데이터, 조사설계 및 내용에 관한 자세한 내용은 국민건강영양조사의 원시자료 소개 페이지(https:// knhanes.kdca.go.kr/knhanes/sub03/sub03_01.do)에서 확인할 수 있다.

연구대상

본 연구의 참여 대상자는 신체구성 검사 및 OA 검사가 모두 이루어진 국민건강영양조사 4기에서 5기에 해당하는 2009-2011년도에 참여한 만 50세 이상의 여성 5,875명을 최초 대상자로 선정하였다. 이후 LMM을 측정하지 않은 1,548명, 무릎 골관절에 대한 방사선 사진이 누락된 1,144명, BMI와 WC가 누락된 16명, 기타 공변량에 대한 데이터가 누락된 324명을 포함하여 총 3,032명의 참가자가 제외되었으며, 최종적으로 총 2,843명의 참가자가 본 연구의 최종 데이터 분석에 포함되었다(Figure 1).

방사선학적 무릎 골관절염의 평가

SD3000 Synchro Stand (Accel Ray, Bern, Switzerland)를 사용하여 양측 무릎이 전후, 외측에서 봤을 때 굽혀진 상태(30° flexion)로 체중이 부하된 자세에서 무릎의 방사선 사진을 촬영하였다. 촬영된 사진은 두 명의 방사선 전문의에 의해 Kellgren-Lawremce (KL) 척도를 통해 평가되었으며, 일치하는 등급을 최종 등급으로 인정하였다. 만일 두 전문의 간 등급의 불일치가 1등급일 경우, 더 높은 등급을 최종 등급으로 인정하였으며, 불일치가 1등급을 초과할 경우 세 번째 방사선 전문의를 통해 평가된 등급과 일치하는 등급을 최종등급으로 인정하였다. 방사선학적 KOA은 KL 등급이 ≥2일 경우로 정의하였다[33].

인체계측 및 하지근육비율, 비만 및 복부비만의 정의

모든 인체계측은 검진용 가운을 착용한 상태에서 진행되었다. 신장은 선 자세에서 신장계(Seca 225, Seca, Hamburg, Germany)를 통해 측정하였으며, WC는 인체계측용 줄자를 통해 늑골 최하단부와 장골의 중앙지점에서 측정하였다. 체성분은 Dual energy X-ray absorptiometry (DXA) 법을 활용한 분석 장비(Discovery-W

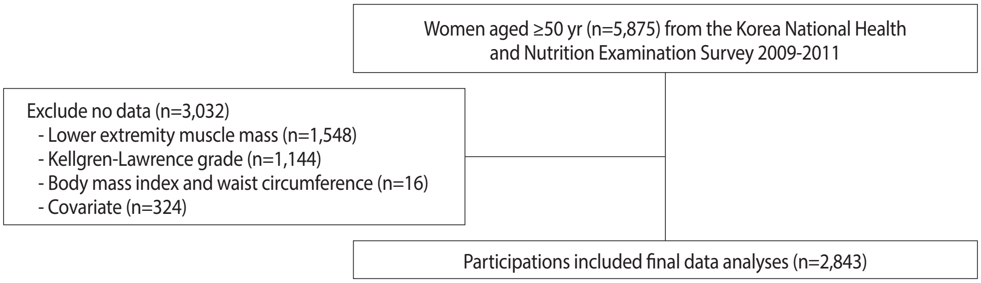

Figure 1. Flow chart of eligible participants in the study.

fan-beam densitometer; Hologic, Bedford, MA, USA)를 통해 측정하였다.

LMM은 DXA를 통해 산출된 다리의 질량에서 뼈와 지방의 질량을 제외하여 산출하였다. 산출된 LMM은 LMM/체중×100의 공식을 대입하여 다리근육비율(lower extremity muscle mass index)로 표준화하였다[34]. 이후 다리근육비율은 사분위수에 근거하여 하위(하위 25%), 중위(중간 50%), 상위(상위 25%)로 분류하였다.

또한 측정된 신장과 체중은 체중(kg)/신장(m^2)의 공식을 통해 BMI로 산출하였으며, 산출된 BMI는 세계보건기구 서태평양지역에서 정의하는 아시아인의 GOB 기준에 따라 25 kg/m^2 이상일 경우 GOB로 정의하였다[10]. 또한 COB는 측정된 WC를 기준으로 대한비만학회의 COB 기준에 근거하여 ≥85 cm일 경우로 정의하였다[35].

공변량

본 연구에서는 공변량으로 연령, WC, 월 평균 가구 소득, 교육 수준, 결혼 상태, 경제활동 상태, 거주 지역, 흡연, 과음, 비신체활동, 에너지섭취량, 단백질 섭취량, 주관적 건강상태, 폐경 유무, 동반질환 수에 대해 조사하였다. 공변량은 자가보고형 설문을 사용하여 평가하였다. 월 평균 가구 소득은 만 원 단위로 산출하였고, 교육수준은 초졸 이하, 중졸 및 고졸, 대졸 이상으로 구분하였으며, 결혼 상태는 기혼과 이혼 및 사별 또는 미혼으로 구분하였다. 경제활동 상태는 현재 소득이 있는 경제활동의 참여 여부에 따라 구분하였으며, 거주 지역은 동과 읍면부로 구분하였다. 흡연은 평생 100개비 이상의 흡연 이력이 있거나 현재 흡연 중인 경우로 정의하였으며[36], 과음은 주 1회 이상 8잔 이상 음주하는 경우로 정의하였다[37]. 비신체활동은 주당 중강도 이상의 신체활동을 150분 이상 참여하지 않는 경우로 정의하였으며[38], 에너지 섭취량과 단백질 섭취량은 하루에 먹는 양에 대해 g/day 단위로 조사하였다. 주관적 건강상태는 매우 좋음 또는 좋음과 보통, 낮음 또는 매우 낮음의 세 범주로 구분하였으며, 동반질환은 의사에게 고혈압, 고지혈증, 당뇨, 뇌졸중, 협심증, 심근경색에 대해 진단받은 수에 따라 0과 1, ≥2로 범주화하였다.

자료 분석

모든 데이터에서 연속형 변수와 범주형 변수는 각각 평균과 표준편차, 요인별 빈도 수 및 비율(%)로 표기하였다. 독립 표본 t 검정과 교차 분석을 사용하여 KOA와 GOB 및 COB 유무에 따른 연속형 변수와 범주형 변수를 비교하였으며, 일원변량분석의 대비다항식과 교차분석의 선형 대 결합을 사용하여 다리근육비율의 범주에 따른 변인들의 경향을 비교하였다. 또한 일부 표본의 랜덤 추출을 통해 측정하는 과정을 여러 번 반복하는 부트스트래핑 방법(n=10,000)에 근거한 매개분석을 이용하여 BMI와 KL 등급, WC와 KL 등급의 연관성에 대한 다리근육비율의 매개효과를 검증하였다. 모든 통계분석은 SPSS-PC 통계 소프트웨어(version 29.0, IBM Corp., Armonk, NY, USA)를 사용하였으며, 추가적으로 매개분석의 경우 SPSS 프로그램 내에 Andrew F. Hayes의 Process macro를 설치하여 분석하였다. 또한 가설 검증을 위한 통계적 유의 수준은 $\alpha = 0.05$로 설정하였다.

연구결과

무릎 골관절염 유무에 따른 변인 비교

Table 1은 KOA 유무에 따른 변인들을 비교한 결과이다. 그 결과 KOA 집단은 정상 집단과 비교하여 연령($p < 0.001$), 체중($p < 0.001$), BMI ($p < 0.001$), WC ($p < 0.001$), 체지방률($p < 0.001$), 교육 수준이 낮은 사람의 비율($p < 0.001$), 주관적 건강 상태가 좋지 않은 사람의 비율($p < 0.001$), 폐경한 사람의 비율($p < 0.001$), 만성질환을 2개 이상 보유한 사람의 비율이 유의하게 높은 것으로 나타났으며($p < 0.001$), 신장($p < 0.001$), 다리근육비율($p < 0.001$), 월 평균 가구소득($p < 0.001$), 기혼 중인 사람의 비율($p < 0.001$), 도시에 거주하는 사람의 비율($p < 0.001$), 과도한 음주를 하는 사람의 비율($p = 0.018$), 에너지 섭취량($p < 0.001$), 단백질 섭취량($p < 0.001$)은 유의하게 낮은 것으로 나타났다.

비만 및 복부비만 유무에 따른 변인 비교

Table 2는 GOB 및 COB 유무에 따른 변인들을 비교한 결과이다. 먼저 GOB 유무에 따른 변인들을 비교한 결과, GOB 집단은

Table 1. Descriptive statistics of measured parameters according to KOA

Variables	Total (n=2,843)	Normal (n=1,477)	KOA (n=1,366)	p-value
KL grade	1.67±1.44	0.42±0.49	3.02±0.75	<0.001
Age (yr)	63.8±9.0	60.4±8.3	67.4±8.3	<0.001
Body weight (kg)	57.0±8.7	56.0±8.1	58.1±9.3	<0.001
Height (cm)	153.2±5.8	154.2±5.6	152.2±5.9	<0.001
LMM (kg)	11.3±1.6	11.2±1.6	11.3±1.7	0.621
LMI (%)	20.0±2.0	20.3±2.0	19.7±2.0	<0.001
BMI (kg/m^2)	24.3±3.3	23.6±3.0	25.0±3.3	<0.001
WC (cm)	82.3±9.3	80.2±8.7	84.7±9.3	<0.001
Body fat (%)	34.7±5.5	34.0±5.4	35.4±5.5	<0.001
Socioeconomic status				
Household income (10,000 KRW/mo)	140.7±246.4	164.5±270.4	115.0±214.6	<0.001
Education				<0.001
Lower than elementary school	1,822 (64.1)	785 (53.1)	1,037 (75.9)	
Middle/high school	886 (31.2)	588 (39.8)	298 (21.8)	
Over than college	135 (4.7)	104 (7.1)	31 (2.3)	
Marital status				<0.001
Married	1,923 (67.6)	1,123 (76.0)	800 (58.6)	
Widowed/divorced/unmarried	920 (32.4)	354 (24.0)	566 (41.4)	
Region of residence				<0.001
Urban	1,947 (68.5)	1,089 (73.7)	858 (62.8)	
Rural	896 (31.5)	388 (26.3)	508 (37.2)	
Health-related parameters				
Smoking	195 (6.9)	100 (6.8)	95 (7.0)	0.846
Binge alcohol	106 (3.7)	54 (3.7)	52 (3.8)	0.832
Inactivity	2,265 (79.7)	1,166 (78.9)	1,099 (80.5)	0.318
Energy intake (g/day)	1,591.1±615.2	1,640.5±615.4	1,537.7±610.7	<0.001
Protein intake (g/day)	53.0±27.6	56.1±29.3	49.6±25.1	<0.001
Self-reported health status				<0.001
Good or very good	765 (26.9)	410 (27.8)	355 (26.0)	
Fair	1,074 (37.8)	607 (41.1)	467 (34.2)	
Poor or very poor	1,004 (35.3)	460 (31.1)	544 (39.8)	
Menopause	2,684 (94.4)	1,353 (91.6)	1,331 (97.4)	<0.001
Comorbidity				<0.001
0	1,305 (45.9)	797 (54.0)	508 (37.2)	
1	932 (32.8)	419 (28.4)	513 (37.6)	
≥2	606 (21.3)	261 (17.6)	345 (25.3)	

Values are presented as mean±standard deviation or number (%).
KOA, knee osteoarthritis; KL, Kellgren-Lawrence; LMM, lower extremity muscle mass; LMI, lower extremity muscle mass index; BMI, body mass index; WC, waist circumference; KRW, Korean won.

정상 집단과 비교하여 WC ($p<0.001$), 체중($p<0.001$), LMM ($p<0.001$), 체지방률($p<0.001$), 교육 수준이 낮은 사람의 비율($p<0.001$), 2가지 이상 만성질환을 보유한 사람의 비율($p<0.001$), KL 등급($p<0.001$), KOA에 해당하는 사람의 비율($p<0.001$)이 유의하게 높은 것으로 나타났으며, 다리근육비율($p<0.001$), 월평균 가구 소득($p=0.016$), 흡연하는 사람의 비율($p=0.014$), 신체활동을 하지 않는 사람의 비율($p=0.003$)은 유의하게 낮은 것으로 나타났다.

또한 COB 유무에 따른 변인들을 비교한 결과, COB 집단은 정상 집단과 비교하여 BMI ($p<0.001$), 연령($p<0.001$), 체중($p<0.001$), 신장($p<0.001$), LMM ($p<0.001$), 체지방률($p<0.001$), 교육 수준이 낮은 사람의 비율($p<0.001$), 주관적 건강상태가 낮은 사람의 비율($p<0.001$), 2개 이상 만성질환을 보유한 사람의 비율($p<0.001$), KL 등급($p<0.001$), KOA에 해당하는 사람의 비율($p<0.001$)이 유의하게 높은 것으로 나타났으며, 다리근육비율($p<0.001$), 월평균 가구소득($p=0.016$), 기혼 중인 사람의 비율($p=0.001$), 단백질 섭취량($p=0.011$)은 유의하게 낮은 것으로 나타났다.

다리근육비율 수준에 따른 변인들의 경향 분석

Table 3은 다리근육비율 수준에 따른 변인들의 경향을 분석한 결과이다. 그 결과 연령($p<0.001$)과 체중($p<0.001$), BMI ($p<0.001$), WC ($p<0.001$), 체지방률($p<0.001$), 교육 수준이 낮은 사람의 비

Table 2. Descriptive statistics of measured parameters according to GOB and COB

Variables	BMI (kg/m²)		p-value	WC (cm)		p-value
	<25 (n=1,763)	≥25 (n=1,080)		<85 (n=1,759)	≥85 (n=1,084)	
BMI (kg/m²)	22.3±1.9	27.5±2.2	<0.001	22.6±2.3	27.0±2.7	<0.001
WC (cm)	77.5±6.8	90.2±7.0	<0.001	76.6±5.8	91.6±5.6	<0.001
Age (yr)	63.8±9.4	63.7±8.4	0.646	63.1±9.3	64.8±8.4	<0.001
Body weight (kg)	52.5±6.1	64.5±7.0	<0.001	52.9±6.5	63.8±7.6	<0.001
Height (cm)	153.4±6.0	153.0±5.6	0.115	152.9±5.9	153.8±5.7	<0.001
LMM (kg)	10.7±1.4	12.1±1.6	<0.001	10.7±1.4	12.1±1.6	<0.001
LMI (%)	20.6±2.0	19.0±1.7	<0.001	20.5±2.0	19.1±1.8	<0.001
Body fat (%)	32.6±5.2	38.1±4.0	<0.001	32.8±5.3	37.7±4.2	<0.001
Socioeconomic status						
Household income (10,000 KRW/mo)	148.3±256.5	128.4±228.5	0.016	149.2±255.8	126.9±229.8	0.016
Education			<0.001			<0.001
Lower than elementary school	1,082 (61.3)	740 (68.5)		1,040 (59.1)	782 (72.1)	
Middle/high school	581 (33.0)	305 (28.3)		611 (34.8)	275 (25.4)	
Over than college	100 (5.7)	35 (3.2)		108 (6.1)	27 (2.5)	
Marital status			0.482			<0.001
Married	1,201 (68.1)	772 (66.9)		1,233 (70.1)	690 (63.7)	
Widowed/divorced/unmarried	562 (31.9)	358 (33.1)		526 (29.9)	394 (36.3)	
Region of residence			0.975			0.090
Urban	1,207 (68.5)	740 (68.5)		1,225 (69.6)	722 (66.6)	
Rural	556 (31.5)	340 (31.5)		534 (30.4)	362 (33.4)	
Health-related parameters						
Smoking	137 (7.8)	58 (5.4)	0.014	125 (7.1)	70 (6.5)	0.506
Binge alcohol	55 (3.1)	51 (4.7)	0.029	53 (3.0)	53 (4.9)	0.010
Inactivity	1,436 (81.5)	829 (76.8)	0.003	1,406 (79.9)	859 (79.2)	0.658
Energy intake (g/day)	1,586.1±638.9	1,599.2±574.5	0.582	1,598.8±637.9	1,578.7±576.4	0.398
Protein intake (g/day)	53.1±27.9	52.8±27.0	0.830	54.0±28.9	51.4±25.2	0.011
Self-reported health status			0.125			<0.001
Good or very good	480 (27.2)	285 (26.4)		501 (28.5)	264 (24.4)	
Fair	685 (38.9)	389 (36.0)		697 (39.6)	377 (34.8)	
Poor or very poor	598 (33.9)	406 (37.6)		561 (31.9)	443 (40.8)	
Menopause	1,670 (94.7)	1,014 (93.9)	0.346	1,650 (93.8)	1,034 (95.4)	0.074
Comorbidity			<0.001			<0.001
0	921 (52.2)	384 (35.5)		948 (53.9)	357 (32.9)	
1	549 (31.1)	383 (35.5)		541 (30.8)	391 (36.1)	
≥2	293 (16.7)	313 (29.0)		270 (15.3)	336 (31.0)	
KL grade	1.43±1.40	2.06±1.44	<0.001	1.41±1.39	2.08±1.43	<0.001
Radiographic KOA	712 (40.4)	654 (60.6)	<0.001	700 (39.8)	666 (61.4)	<0.001

Values are presented as mean±standard deviation or number (%).
GOB, general obesity; COB, central obesity; BMI, body mass index; WC, waist circumference; LMM, lower extremity muscle mass; LMI, lower extremity muscle mass index; KRW, Korean won; KL, Kellgren-Lawrence; KOA, knee osteoarthritis.

율($p<0.001$), 기혼 중인 사람의 비율($p=0.007$), 도시에 거주하는 사람의 비율($p=0.017$), 주관적 건강상태가 좋지 않은 사람의 비율($p=0.008$), 2개 이상의 만성질환을 보유한 사람의 비율($p<0.001$), KL 등급($p<0.001$), KOA에 해당하는 사람의 비율($p<0.001$)은 다리근육비율 수준이 높아질수록 낮아지는 경향이 있는 것으로 나타났으며, 신장($p<0.001$)과 LMM ($p<0.001$), 에너지 섭취량($p<0.001$)은 다리근육비율 수준이 높아질수록 높아지는 경향이 있는 것으로 나타났다.

체질량지수와 KL 등급, 허리둘레와 KL 등급의 연관성에 대한 다리근육비율의 매개 분석

Figure 2A와 Table 4는 다리근육비율에 의해 매개되는 BMI와 KL 등급, WC와 KL 등급의 연관성을 보여준다. 먼저 BMI와 KL 등급의 연관성에 대한 다리근육비율의 매개효과 분석을 위해 실시한 결과, BMI는 KL 등급에 직접적인 영향을 미치는 것으로 나타났으며($ß=0.092$, $p<0.001$, c` path), 다리근육비율에 대한 영향을 통해 KL 등급에 간접적인 영향을 미치는 것으로 나타났다 (Figure 2A, Table 4). BMI는 KL 등급과 음의 연관성을 나타내는

다리근육비율(ß = -0.048, p = 0.001, b path)과 음의 연관성을 보이는 것으로 나타났다(ß = -0.320, p < 0.001, a path). 또한 측정된 모든 공변량에 대한 조정 후에도 BMI와 다리근육비율은 KL 등급의 중요한 결정 요인으로 나타났다(ß = -0.034, p = 0.037, b path; ß = 0.103, p < 0.001, c` path).

부트스트래핑을 통해 BMI와 KL 등급 간의 연관성에 대한 다리근육비율의 매개 효과를 분석한 결과, 95% 신뢰구간(0.006-0.025)이 0을 포함하지 않는 것으로 나타나 다리근육비율이 BMI와 KL 등급 간의 연관성을 중재함을 의미하는 결과가 도출되었으며, KL 등급에 대한 BMI의 전체 효과 중 14.0%가 다리근육비율을 통해 설명되는 것으로 확인되었다. BMI와 KL 등급의 연관성에 대한 다리근육비율의 매개 효과는 공변량에 대한 조정 후에도 유의하게 나타났으며(95% CI: 0.000-0.010), 전체 효과 중 4.6%가 다리근육비율을 통해 설명되었다. 이러한 결과는 GOB가 KOA의 발병에 직접적으로 영향을 미치지만 다리근육을 통해 간접적으로도 영향을 미치는 것으로 해석된다.

마찬가지로 WC와 KL 등급의 연관성에 대한 다리근육비율의 매개 효과 분석을 실시한 결과, WC는 KL 등급에 직접적인 영향

Table 3. Descriptive statistics of measured parameters according to LMI

Variables	Lower LMI (n=711, 25.0%)	Middle LMI (n=1,421, 50.0%)	Upper LMI (n=711, 25.0%)	p for trend
LMI (%)	17.5±0.9	19.9±0.7	22.7±1.2	<0.001
Age (yr)	65.5±8.8	63.2±8.8	63.4±9.4	<0.001
Body weight (kg)	60.7±9.1	57.4±8.0	52.7±7.8	<0.001
Height (cm)	151.8±5.5	153.5±5.7	154.1±6.3	<0.001
LMM (kg)	10.6±1.6	11.3±1.6	11.8±1.6	<0.001
BMI (kg/m²)	26.3±3.4	24.3±2.8	22.1±2.6	<0.001
WC (cm)	87.5±9.4	82.4±8.2	76.9±8.0	<0.001
Body fat (%)	40.2±3.4	34.9±3.2	28.7±4.6	<0.001
Socioeconomic status				
Household income (10,000 KRW/mo)	240.1±9.0	230.1±6.1	281.3±10.5	0.238
Education				<0.001
Lower than elementary school	504 (70.9)	890 (62.6)	428 (60.2)	
Middle/high school	185 (26.0)	462 (32.5)	239 (33.6)	
Over than college	22 (3.1)	69 (4.9)	44 (6.2)	
Marital status				0.007
Married	438 (61.6)	999 (70.3)	486 (68.4)	
Widowed/divorced/unmarried	273 (38.4)	422 (29.7)	225 (31.6)	
Region of residence				0.017
Urban	489 (68.8)	1,011 (71.1)	447 (62.9)	
Rural	222 (31.2)	410 (28.9)	264 (37.1)	
Health-related parameters				
Smoking	42 (5.9)	98 (6.9)	55 (7.7)	0.173
Binge alcohol	28 (3.9)	54 (3.8)	24 (3.4)	0.576
Inactivity	572 (80.5)	1,141 (80.3)	552 (77.6)	0.188
Energy intake (g/day)	1,508.7±556.2	1,607.2±631.5	1,641.4±630.9	<0.001
Protein intake (g/day)	51.0±28.1	53.7±26.9	53.6±28.2	0.075
Self-reported health status				0.008
Good or very good	171 (24.1)	397 (27.9)	197 (27.7)	
Fair	246 (34.6)	556 (39.1)	272 (38.3)	
Poor or very poor	294 (41.3)	468 (33.0)	242 (34.0)	
Menopause	687 (96.6)	1,325 (93.2)	672 (94.5)	0.083
Comorbidity				<0.001
0	249 (35.0)	630 (44.3)	426 (59.9)	
1	249 (35.0)	487 (34.3)	196 (27.6)	
≥2	213 (30.0)	304 (21.4)	89 (12.5)	
KL grade	1.52±0.06	1.41±0.04	1.34±0.05	<0.001
Radiographic KOA	423 (59.5)	660 (46.4)	283 (39.8)	<0.001

Values are presented as mean±standard deviation or number (%).
LMI, lower extremity muscle mass index; LMM, lower extremity muscle mass; BMI, body mass index; WC, waist circumference; KRW, Korean won; KL, Kellgren-Lawrence; KOA, knee osteoarthritis.

을 미치는 것으로 나타났으며(ß = 0.036, p < 0.001, c` path), 다리근육비율에 대한 영향을 통해서도 KL 등급에 간접적인 영향을 미치는 것으로 나타났다(Figure 2B, Table 4). WC는 KL 등급과 음의 연관성을 나타내는 다리근육비율(ß = -0.049, p = 0.001, b path)과 음의 연관성을 보이는 것으로 나타났다(ß = -0.100, p < 0.001, a path). 또한 측정된 공변량에 대한 조정 후에도 WC와 다리근육비율은 KL 등급의 중요한 결정 요인으로 나타났다(ß = -0.051, p = 0.002, b path; ß = 0.029, p < 0.001, c` path).

부트스트래핑을 통해 WC와 KL 등급 간의 연관성에 대한 다리근육비율의 매개 효과를 분석한 결과, 95% 신뢰구간(0.002-0.008)이 0을 포함하지 않는 것으로 나타나 다리근육비율이 WC와 KL 등급 간의 연관성을 중재함을 의미하는 결과가 도출되었으며, KL 등급에 대한 WC의 전체 효과 중 12.2%가 다리근육비율을 통해 설명되는 것으로 확인되었다. WC와 KL 등급의 연관성에 대한 다리근육비율의 매개 효과는 공변량에 대한 조정 후에도 유의하게 나타났으며(95% CI, 0.001-0.003), 전체 효과 중 6.5%가 다리근육비율을 통해 설명되었다. 이러한 결과는 COB가 KOA의 발병에 직접적으로 영향을 미치지만 다리근육을 통해 간접적으로도 영향을 미치는 것으로 해석된다.

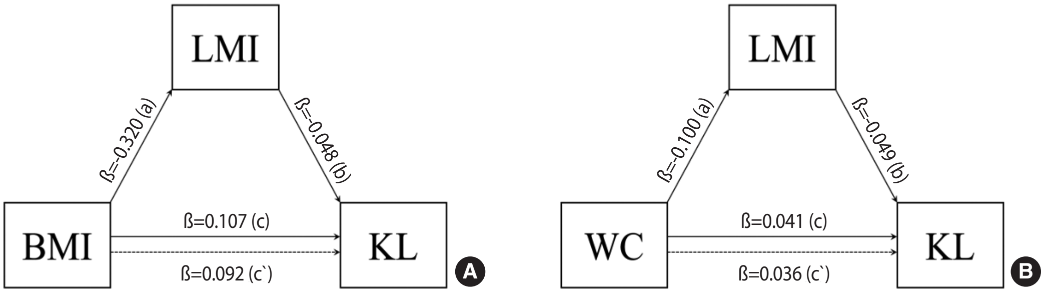

Figure 2. Mediation analysis. Path coefficients of body mass index (BMI; A) or waist circumference (WC; B) on Kellgren-Lawrence grade (KL) through lower extremity muscle mass index (LMI). Path a: estimated coefficient for the regression with BMI (A) or WC (B) predicting LMI; Path b: estimated coefficient for the regression with LMI predicting KL; Path c=total effects for the regression with BMI (A) or WC (B) predicting KL; Path c`=direct effects for the regression with BMI (A) or WC (B) predicting KL independent of LMI.

Table 4. The association between obesity and KL, mediated by LMI, in Korean women aged over 50 years[1]

Path[2]	Model 1				Model 2			
	ß (SE)	95% CI		p-value	ß (SE)	95% CI		p-value
		UL	LL			UL	LL	
General obesity								
BMI→LMI, a	-0.320 (0.010)	-0.340	-0.300	<0.001	-0.147 (0.010)	-0.166	-0.127	<0.001
LMI→KL, b	-0.048 (0.015)	-0.077	-0.019	0.001	-0.032 (0.016)	-0.064	-0.001	0.046
Total effect, c	0.107 (0.008)	0.092	0.123	<0.001	0.105 (0.009)	0.088	0.122	<0.001
Direct effect, c`	0.092 (0.009)	0.074	0.111	<0.001	0.100 (0.009)	0.082	0.118	<0.001
Indirect effect, ab	0.015 (0.005)	0.006	0.025		0.005 (0.003)	0.000	0.010	
Indirect to total effect (%)	14.0				4.8			
Central obesity								
WC→LMI, a	-0.100 (0.004)	-0.107	-0.093	<0.001	-0.040 (0.004)	-0.047	-0.033	<0.001
LMI→KL, b	-0.049 (0.014)	-0.077	-0.021	0.001	-0.049 (0.016)	-0.081	-0.017	0.002
Total effect, c	0.041 (0.003)	0.035	0.046	<0.001	0.030 (0.003)	0.024	0.036	<0.001
Direct effect, c`	0.036 (0.003)	0.030	0.042	<0.001	0.028 (0.003)	0.022	0.034	<0.001
Indirect effect, ab	0.005 (0.001)	0.002	0.008		0.002 (0.001)	0.001	0.003	
Indirect to total effect (%)	12.2				6.7			

KL, Kellgren-Lawrence grade; LMI, lower extremity muscle mass index; SE, standard error; CI, confidence interval; UL, upper limit; LL, lower limit; BMI, body mass index; WC, waist circumference.
[1]Model 1: Non-adjusted; Model 2: Adjusted for age, body fat, household income, education, marital status, region, smoking, binge alcohol, inactivity, energy intake, protein intake, self-reported health status, menopause, comorbidity.
[2]In the mediation model, the indirect effect is the product of path coefficients a (BMI or WC→LMI) and b (LMI→KL); The direct effect is the coefficient c`; The total effect (c) is equal to the sum of the direct and indirect (c` + ab).

고 찰

본 연구에서는 한국의 50세 이상에 해당하는 중·고령 여성 2,843명을 대상으로 GOB 및 COB와 KOA의 관계에 대한 다리근육비율의 매개효과를 조사한 결과, GOB를 평가하는 BMI와 COB를 평가하는 WC, 그리고 다리근육비율 모두 KOA를 진단하는 KL 등급의 중요한 예측인자로 나타났다. 또한 KL 등급에 대한 BMI, KL 등급에 대한 WC의 영향은 다리근육비율에 의해 부분적으로 매개되는 것으로 나타났다.

GOB에 기인하여 무릎 관절에 가해지는 과부하는 응력을 초과하여 관절 연골[39-42]과 골 연골의 인터페이스[43], 반월판[44]에서 KOA의 발병을 촉진하는 병리학적 손상을 야기한다. 또한 지방세포에서 분비되는 염증전 사이토카인의 일부는 관절을 분해하는 대사 과정을 유도하여 OA의 발병을 촉진하는 것으로 보고된다[45]. 이에 과도한 체중과 지방의 축적을 특징으로 하는 GOB와 COB는 KOA의 발병에 인과적 역할을 하는 것으로 알려져 있으며, 이는 많은 역학연구들을 통해 인종과 성별, 연령 등과 무관한 KOA 발병의 주요 위험 요인으로 잘 확립되어있다[11-15,23-25]. BMI 및 WC와 KL 등급 간 유의한 정적 연관성이 나타난 본 연구의 결과는 이전의 연구결과와 일치한다.

다리의 근육은 외부 하중에 대응하기 위한 힘을 생성하여 무릎 관절에 가해지는 부하를 흡수하고, 동적 상황에서 관절에 안정성을 제공한다[20]. 이러한 무릎 관절에 대한 다리 근육의 보호 효과는 무릎 관절의 안정성에 대한 다리 근육의 기여도를 평가한 실험 연구를 통해 무릎 관절을 둘러싸고 있는 특정 근육만이 기여하는 것이 아닌[46], 다리에 위치한 대부분의 근육들이 각기 다른 방식으로 기여하는 것으로 확인된다[47]. 이는 즉, 전반적으로 다리 근력이 우수할수록 KOA의 발병으로부터 강한 보호 효과를 발휘할 수 있음을 의미한다. 따라서 근력이 근육량과 비례함을 감안할 때, LMM은 다리에 위치한 근육들의 힘을 반영하는 것으로 추측되며[48], 다리의 근력과 마찬가지로 KOA의 발병으로부터 보호 효과를 나타낼 것으로 판단된다. 실례로, 한국 성인의 대표 표본에서 LMM을 체중으로 나눈 다리근육비율은 KOA의 발병 위험과 유의한 음의 연관성을 나타내 이를 뒷받침하였다[31,32]. 그러나 Segal et al. [49]의 코호트 연구에서 허벅지 근육량은 KOA의 발병으로부터 보호 효과를 제공하지 못하는 것으로 나타났다. 이와 같은 상반된 선행연구의 결과는 체중을 고려한 LMM의 상대값과 절대값 사용에 따른 차이로 판단되며, 일반적으로 지방량, 근육량과 같은 신체구성이 체중과 비례함을 감안할 때, 이러한 영향을 보정할 수 있는 LMM에 대한 상대값의 사용이 다리 근력을 보다 명확히 반영할 것으로 예상된다. 본 연구의 경우 다리근육비율과 KL 등급 간 유의한 음의 연관성이 나타나 LMM을 체중으로 보정한 다리근육비율이 KOA의 발병으로부터 보호 효과를 제공한다고 보고한 이전의 연구[31,32]와 일치하는 결과가 도출되었으며, 종합적으로 이러한 결과들은 체중 대비 많은 다리의 근육량이 KOA의 발병으로부터 보호 역할을 제공할 수 있음을 시사한다.

한편, 본 연구는 BMI와 KL 등급, WC와 KL 등급의 연관성이 다리근육비율에 의해 부분적으로 매개된다는 보고를 한 최초의 연구로, 다리근육비율의 부분 매개 효과는 인구통계학적 요인과 사회경제적 요인, 건강 관련 매개 변수 등 잠재적 공변량과 무관하게 유의한 것으로 나타났다. 이와 유사한 연구로 한국의 2010-2011 KNHANES에 참여한 4,194명의 데이터를 분석한 Kim et al. [31]의 연구에서는 정상 및 GOB 집단에서 LMM이 많을수록 KOA의 발병 위험이 감소하는 선경향이 관찰되었으며, 또한 이 연구에서 GOB더라도 다리 근육이 많은 집단이 LMM이 적은 집단보다 조사된 모든 공변량과 무관하게 KOA의 발병 위험이 유의하게 낮은 것으로 나타났다. 이러한 선행연구와 본 연구의 결과는 다리 근육 강화가 KOA에 대한 GOB 및 COB의 영향을 완화할 수 있음을 암시하며, 건강한 생활습관과 규칙적인 근력운동의 실천을 통해 다리 근육을 강화하는 것이 KOA의 예방을 위한 전략에 포함되어야 함을 시사한다. 다만 본 연구에서 BMI 및 WC와 KOA의 연관성에 대한 다리근육비율의 매개효과는 각각 4.6%와 6.5%로 미미하였으며, 이를 고려할 때, KOA의 예방에 있어 GOB 및 COB의 관리가 다리근육의 강화보다 중점적으로 선행되어야 할 것으로 판단된다.

본 연구에서 나타난 GOB 및 COB와 KOA의 연관성에 대한 다리근육비율의 매개 효과를 토대로 다리근육비율은 GOB 및 COB로 인한 KOA의 발병으로부터 보호 효과를 제공할 수 있으리라 예상되며, 이에 대해 다음과 같은 설명이 제공될 수 있다. 먼저 생체역학적 관점에서 GOB 및 COB는 무릎 관절에 비정상적인 스트레스를 가하여 관절의 안정성을 손상시킨다. 반면 근육은 관절 기능 중에 부하되는 충격을 흡수하여 관절의 안정성 유지에 기여하는 역할을 한다[20]. 따라서 GOB나 COB는 인해 무릎 관절에 가해지는 비정상적인 스트레스를 다리 근육이 완충할 수 있으리라 예상되며, 본 연구와 근감소증과 GOB를 동반하는 근감소성 GOB가 GOB보다 KOA의 위험이 더 높다고 보고한 선행연구는 이러한 설명을 뒷받침한다[50].

다만 현재 연구에는 몇 가지 한계가 있다. 첫째, 연구의 결과는 인종이나 연령, 성별에 따라 차이가 있을 수 있다. 그러나 본 연구에서 한국의 50세 미만의 남녀와 50세 이상의 남성은 한국 인구의 특성상 낮은 KOA의 유병률로 인해 제외하였으며, 한국의 50세 이상 성인 여성으로 대상이 국한되었다. 따라서 결과는 다른 민족 인구나 한국의 남성, 50세 이상 미만의 여성으로 일반화할 수 없다. 둘째, KNHANES 조사에는 복용 중인 약물, 대사 위험 요인, 호르몬 등 KOA에 영향을 미칠 수 있는 교란 요인에 대한 정보가 제공되지 않기에 조사되지 않은 잠재적 공변량이 결과에 영

향을 미칠 가능성을 배제할 수 없다. 셋째, KNHANES 조사에서는 인공관절 수술 여부를 알 수 있는 정보가 제공되지 않는다. 따라서 인공관절 수술을 시행한 사람을 연구대상에서 배제하지 못하였을 가능성이 존재한다. 넷째, 본 연구는 횡단 설계된 연구로 연구결과에 대한 인과 관계를 설명하지 못한다. 마지막으로 본 연구는 다리 근육이 GOB 및 COB와 KOA의 관계를 매개하는 생리학적 기전을 제공하지 못하며, 따라서 이를 확인하기 위한 추가 연구가 필요하다.

결론적으로 본 연구의 결과는 한국의 50세 이상 여성의 BMI 및 WC와 KOA의 관계에 대한 다리근육비율의 매개 효과를 나타낸다. 이러한 결과는 다리 근육의 강화가 GOB 및 COB로 인한 KOA의 위험을 부분적으로 완화시킬 수 있음을 암시하며, 건강한 생활습관과 규칙적인 근력운동의 병행을 통해 GOB 및 COB 관리와 더불어 다리 근육을 강화하는 것이 KOA의 예방을 위한 전략으로 권장되어야 함을 시사한다.

참고문헌

1. Kanamoto T, Mae T, Yokoyama T, Tanaka H, Ebina K, Nakata K. Significance and definition of early knee osteoarthritis. Ann Jt 2020;5:4. https://doi.org/10.21037/aoj.2019.09.02
2. Jang S, Lee K, Ju JH. Recent updates of diagnosis, pathophysiology, and treatment on osteoarthritis of the knee. Int J Mol Sci 2021;22:2619. https://doi.org/10.3390/ijms22052619
3. GBD 2015 Disease and Injury Incidence and Prevalence Collaborators. Global, regional, and national incidence, prevalence, and years lived with disability for 310 diseases and injuries, 1990-2015: a systematic analysis for the Global Burden of Disease Study 2015. Lancet 2016;388:1545-1602. https://doi.org/10.1016/S0140-6736(16)31678-6
4. Cui A, Li H, Wang D, Zhong J, Chen Y, Lu H. Global, regional prevalence, incidence and risk factors of knee osteoarthritis in population-based studies. EClinicalMedicine 2020;29-30:100587. https://doi.org/10.1016/j.eclinm.2020.100587
5. Bannuru RR, Osani MC, Vaysbrot EE, Arden NK, Bennell K, Bierma-Zeinstra SM, et al. OARSI guidelines for the non-surgical management of knee, hip, and polyarticular osteoarthritis. Osteoarthritis Cartilage 2019;27:1578-1589. https://doi.org/10.1016/j.joca.2019.06.011
6. Losina E, Paltiel AD, Weinstein AM, Yelin E, Hunter DJ, Chen SP, et al. Lifetime medical costs of knee osteoarthritis management in the United States: impact of extending indications for total knee arthroplasty. Arthritis Care Res (Hoboken) 2015;67:203-215. https://doi.org/10.1002/acr.22412
7. Roos EM, Arden NK. Strategies for the prevention of knee osteoarthritis. Nat Rev Rheumatol 2016;12:92-101. https://doi.org/10.1038/nrrheum.2015.135
8. Mahmoudian A, Van Assche D, Herzog W, Luyten FP. Towards secondary prevention of early knee osteoarthritis. RMD Open 2018;4:e000468. https://doi.org/10.1136/rmdopen-2017-000468
9. Whittaker JL, Runhaar J, Bierma-Zeinstra S, Roos EM. A lifespan approach to osteoarthritis prevention. Osteoarthritis Cartilage 2021;29:1638-1653. https://doi.org/10.1016/j.joca.2021.06.015
10. World Health Organization. The Asia-Pacific perspective: redefining obesity and its treatment; 2000 [cited 2023 Oct 19]. Available from: https://apps.who.int/iris/bitstream/handle/10665/206936/0957708211_eng.pdf
11. Coggon D, Reading I, Croft P, McLaren M, Barrett D, Cooper C. Knee osteoarthritis and obesity. Int J Obes Relat Metab Disord 2001;25:622-627. https://doi.org/10.1038/sj.ijo.0801585
12. Raud B, Gay C, Guiguet-Auclair C, Bonnin A, Gerbaud L, Pereira B, et al. Level of obesity is directly associated with the clinical and functional consequences of knee osteoarthritis. Sci Rep 2020;10:3601. https://doi.org/10.1038/s41598-020-60587-1
13. Lee R, Kean WF. Obesity and knee osteoarthritis. Inflammopharmacology 2012;20:53-58. https://doi.org/10.1007/s10787-011-0118-0
14. Felson DT, Anderson JJ, Naimark A, Walker AM, Meenan RF. Obesity and knee osteoarthritis. The Framingham Study. Ann Intern Med 1988;109:18-24. https://doi.org/10.7326/0003-4819-109-1-18
15. Sowers MR, Karvonen-Gutierrez CA. The evolving role of obesity in knee osteoarthritis. Curr Opin Rheumatol 2010;22:533-537. https://doi.org/10.1097/BOR.0b013e32833b4682
16. Liu M, Jin F, Yao X, Zhu Z. Disease burden of osteoarthritis of the knee and hip due to a high body mass index in China and the USA: 1990-2019 findings from the Global Burden of Disease Study 2019. BMC Musculoskelet Disord 2022;23:63. https://doi.org/10.1186/s12891-022-05027-z
17. Spitaels D, Mamouris P, Vaes B, Smeets M, Luyten F, Hermens R, et al. Epidemiology of knee osteoarthritis in general practice: a registry-based study. BMJ Open 2020;10:e031734. https://doi.org/10.1136/bmjopen-2019-031734
18. Smith KB, Smith MS. Obesity statistics. Prim Care 2016;43:121-135. https://doi.org/10.1016/j.pop.2015.10.001
19. Long H, Liu Q, Yin H, Wang K, Diao N, Zhang Y, et al. Prevalence trends of site-specific osteoarthritis from 1990 to 2019: findings

from the Global Burden of Disease Study 2019. Arthritis Rheumatol 2022;74:1172-1183. https://doi.org/10.1002/art.42089
20. Bennell KL, Wrigley TV, Hunt MA, Lim BW, Hinman RS. Update on the role of muscle in the genesis and management of knee osteoarthritis. Rheum Dis Clin North Am 2013;39:145-176. https://doi.org/10.1016/j.rdc.2012.11.003
21. Matheny JB, Goff MG, Pownder SL, Koff MF, Hayashi K, Yang X, et al. An in vivo model of a mechanically-induced bone marrow lesion. J Biomech 2017;64:258-261. https://doi.org/10.1016/j.jbiomech.2017.09.020
22. Wu P, Holguin N, Silva MJ, Fu M, Liao W, Sandell LJ. Early response of mouse joint tissue to noninvasive knee injury suggests treatment targets. Arthritis Rheumatol 2014;66:1256-1265. https://doi.org/10.1002/art.38375
23. Sun Y, Li Y, Yu T, Zhang J. Causal associations of anthropometric measurements with osteoarthritis: a Mendelian randomization study. PLoS One 2023;18:e0279198. https://doi.org/10.1371/journal.pone.0279198
24. Betancourt MC, Marchi E, Lipay M. Associations of central obesity with earlier onset of osteoarthritis symptoms. Osteoarthr Cartil 2021;29(Suppl 1):S20-S21. https://doi.org/10.1016/j.joca.2021.02.043
25. Park D, Park YM, Ko SH, Hyun KS, Choi YH, Min DU, et al. Association of general and central obesity, and their changes with risk of knee osteoarthritis: a nationwide population-based cohort study. Sci Rep 2023;13:3796. https://doi.org/10.1038/s41598-023-30727-4
26. Lee SY, Tung HH, Liu CY, Chen LK. Physical activity and sarcopenia in the geriatric population: a systematic review. J Am Med Dir Assoc 2018;19:378-383. https://doi.org/10.1016/j.jamda.2018.02.003
27. Oussaada SM, van Galen KA, Cooiman MI, Kleinendorst L, Hazebroek EJ, van Haelst MM, et al. The pathogenesis of obesity. Metabolism 2019;92:26-36. https://doi.org/10.1016/j.metabol.2018.12.012
28. Hopkins M, Blundell JE. Energy balance, body composition, sedentariness and appetite regulation: pathways to obesity. Clin Sci (Lond) 2016;130:1615-1628. https://doi.org/10.1042/CS20160006
29. Øiestad BE, Juhl CB, Eitzen I, Thorlund JB. Knee extensor muscle weakness is a risk factor for development of knee osteoarthritis. A systematic review and meta-analysis. Osteoarthritis Cartilage 2015;23:171-177. https://doi.org/10.1016/j.joca.2014.10.008
30. Patterson BE, Girdwood MA, West TJ, Bruder AM, Øiestad BE, Juhl C, et al. Muscle strength and osteoarthritis of the knee: a systematic review and meta-analysis of longitudinal studies. Skeletal Radiol 2023;52:2085-2097. https://doi.org/10.1007/s00256-022-04266-4
31. Kim SR, Choi KH, Jung GU, Shin D, Kim K, Park SM. Associations between fat mass, lean mass, and knee osteoarthritis: the fifth Korean National Health and Nutrition Examination Survey (KNHANES V). Calcif Tissue Int 2016;99:598-607. https://doi.org/10.1007/s00223-016-0190-y
32. Suh DH, Han KD, Hong JY, Park JH, Bae JH, Moon YW, et al. Body composition is more closely related to the development of knee osteoarthritis in women than men: a cross-sectional study using the Fifth Korea National Health and Nutrition Examination Survey (KNHANES V-1, 2). Osteoarthritis Cartilage 2016;24:605-611. https://doi.org/10.1016/j.joca.2015.10.011
33. Kellgren JH, Lawrence JS. Radiological assessment of osteo-arthrosis. Ann Rheum Dis 1957;16:494-502. https://doi.org/10.1136/ard.16.4.494
34. Cheon YH, Kim HO, Suh YS, Kim MG, Yoo WH, Kim RB, et al. Relationship between decreased lower extremity muscle mass and knee pain severity in both the general population and patients with knee osteoarthritis: findings from the KNHANES V 1-2. PLoS One 2017;12:e0173036. https://doi.org/10.1371/journal.pone.0173036
35. Lee SY, Park HS, Kim DJ, Han JH, Kim SM, Cho GJ, et al. Appropriate waist circumference cutoff points for central obesity in Korean adults. Diabetes Res Clin Pract 2007;75:72-80. https://doi.org/10.1016/j.diabres.2006.04.013
36. Centers for Disease Control and Prevention (CDC). Cigarette smoking among adults--United States, 1992, and changes in the definition of current cigarette smoking. MMWR Morb Mortal Wkly Rep 1994;43:342-346.
37. Korea Disease Control and Prevention Agency. Guidebook for data users (2010-2012) of Korea National Health and Nutrition Examination Survey; 2012 [cited 2024 Jan 3]. Available from: https://knhanes.kdca.go.kr/knhanes/sub03/sub03_02_05.do (Korean).
38. Bull FC, Al-Ansari SS, Biddle S, Borodulin K, Buman MP, Cardon G, et al. World Health Organization 2020 guidelines on physical activity and sedentary behaviour. Br J Sports Med 2020;54:1451-1462. https://doi.org/10.1136/bjsports-2020-102955
39. Anandacoomarasamy A, Smith G, Leibman S, Caterson I, Giuffre B, Fransen M, et al. Cartilage defects are associated with physical disability in obese adults. Rheumatology (Oxford) 2009;48:1290-1293. https://doi.org/10.1093/rheumatology/kep246
40. Ding C, Cicuttini F, Scott F, Cooley H, Jones G. Knee structural

alteration and BMI: a cross-sectional study. Obes Res 2005;13:350-361. https://doi.org/10.1038/oby.2005.47

41. Widmyer MR, Utturkar GM, Leddy HA, Coleman JL, Spritzer CE, Moorman CT 3rd, et al. High body mass index is associated with increased diurnal strains in the articular cartilage of the knee. Arthritis Rheum 2013;65:2615-2622. https://doi.org/10.1002/art.38062
42. Kaspiris A, Khaldi L, Chronopoulos E, Vasiliadis E, Grivas TB, Kouvaras I, et al. Macrophage-specific metalloelastase (MMP-12) immunoexpression in the osteochondral unit in osteoarthritis correlates with BMI and disease severity. Pathophysiology 2015;22:143-151. https://doi.org/10.1016/j.pathophys.2015.06.001
43. Chen L, Yao F, Wang T, Li G, Chen P, Bulsara M, et al. Horizontal fissuring at the osteochondral interface: a novel and unique pathological feature in patients with obesity-related osteoarthritis. Ann Rheum Dis 2020;79:811-818. https://doi.org/10.1136/annrheumdis-2020-216942
44. Rai MF, Sandell LJ, Cheverud JM, Brophy RH. Relationship of age and body mass index to the expression of obesity and osteoarthritis-related genes in human meniscus. Int J Obes (Lond) 2013;37:1238-1246. https://doi.org/10.1038/ijo.2012.221
45. Sokolove J, Lepus CM. Role of inflammation in the pathogenesis of osteoarthritis: latest findings and interpretations. Ther Adv Musculoskelet Dis 2013;5:77-94. https://doi.org/10.1177/1759720X12467868
46. Sritharan P, Lin YC, Pandy MG. Muscles that do not cross the knee contribute to the knee adduction moment and tibiofemoral compartment loading during gait. J Orthop Res 2012;30:1586-1595. https://doi.org/10.1002/jor.22082
47. Buchanan TS, Lloyd DG. Muscle activation at the human knee during isometric flexion-extension and varus-valgus loads. J Orthop Res 1997;15:11-17. https://doi.org/10.1002/jor.1100150103
48. Zhang X, Pan X, Deng L, Fu W. Relationship between knee muscle strength and fat/muscle mass in elderly women with knee osteoarthritis based on dual-energy X-ray absorptiometry. Int J Environ Res Public Health 2020;17:573. https://doi.org/10.3390/ijerph17020573
49. Segal NA, Findlay C, Wang K, Torner JC, Nevitt MC. The longitudinal relationship between thigh muscle mass and the development of knee osteoarthritis. Osteoarthritis Cartilage 2012;20:1534-1540. https://doi.org/10.1016/j.joca.2012.08.019
50. Lee S, Kim TN, Kim SH. Sarcopenic obesity is more closely associated with knee osteoarthritis than is nonsarcopenic obesity: a cross-sectional study. Arthritis Rheum 2012;64:3947-3954. https://doi.org/10.1002/art.37696
